# HIV/AIDS, chronic diseases and globalisation

**DOI:** 10.1186/1744-8603-7-31

**Published:** 2011-08-26

**Authors:** Christopher J Colvin

**Affiliations:** 1 Centre for Infectious Disease Epidemiology and Research (CIDER), Falmouth 5.49, UCT Med School Campus, School of Public Health and Family Medicine, University of Cape Town, Observatory, Cape Town, 7925, South Africa

## Abstract

HIV/AIDS has always been one of the most thoroughly global of diseases. In the era of widely available anti-retroviral therapy (ART), it is also commonly recognised as a chronic disease that can be successfully managed on a long-term basis. This article examines the chronic character of the HIV/AIDS pandemic and highlights some of the changes we might expect to see at the global level as HIV is increasingly normalised as "just another chronic disease". The article also addresses the use of this language of chronicity to interpret the HIV/AIDS pandemic and calls into question some of the consequences of an uncritical acceptance of concepts of chronicity.

## Background

HIV/AIDS has always been one of the most thoroughly global of diseases. From its still hazily understood emergence as a zoonotic infection in colonial and post-colonial West and Central Africa and the early moral panics over a globe-trotting "Patient Zero" to the current situation of global pandemic, it has always been intimately bound up in globalised structures and processes [1-3].

If HIV was global from its beginnings, it came to be seen as chronic only shortly thereafter. In 1989, soon after the development of the first anti-retroviral monotherapies to treat AIDS, Samuel Broder, head of the US National Cancer Institute, famously asserted at an international AIDS conference that HIV should be considered to be a chronic illness and its treatment "should follow the model of cancer". The 1992 book *AIDS: The Making of a Chronic Disease *[4] provided an historical account of HIV activism, clinical treatment, and pharmaceutical research in the 80s that transformed the disease from an acute and consistently fatal condition to one that promised to be manageable over the long term through drug therapy.

From this initial period of the first life-extending treatments in the late 80s to the triple therapy cocktails of the late 90s and now, in the era of large-scale, public-sector ART programmes, HIV clinicians and activists have consistently pushed for a recognition of HIV as "just another chronic disease" [5]. These attempts to characterise HIV as a chronic--and by implication, a stable, manageable, even normal--infection, however, have also always existed in tension with efforts to exceptionalise the epidemic. On the one hand, when treatment became available, activists and clinicians sought to convince patients that HIV was no longer a death sentence. On the other hand, there was real resistance to the normalisation implied in such comparisons with chronic diseases like diabetes. There has been a consistent push to maintain the special status of HIV as a unique global health challenge even as its identity as a chronic condition gains strength [6,7].

What does HIV/AIDS' status as one of the most prominent global and increasingly chronic diseases have to tell us about the broader questions raised in this special issue about the place of chronic diseases and the idea of chronicity in global health? This article examines the intersection of globalisation, the HIV/AIDS pandemic, and the idea of chronicity. It highlights recent shifts in the character of the global HIV/AIDS epidemic and asks how its increasingly chronic nature might be changing global understandings of and responses to the disease. It also argues that conventional notions of chronicity are often inadequate to capture the complexities of not only HIV/AIDS but many of the other diseases routinely interpreted as chronic as well.

### How is the Global HIV Epidemic Changing?

For the last 30 years, the world's response to HIV has gone through a number of dramatic transformations including the rise of global AIDS activism and institutions, the development of effective anti-retroviral therapies, and struggles against several varieties of AIDS denialism [8,9]. There are a number of other, more recent developments in the global epidemic, however, especially in those countries with the highest burden of HIV, that are vital to understand.

Over the last ten years, in high-prevalence countries like those in Southern Africa, increasing (and increasingly visible) AIDS-related mortality, mass prevention and education campaigns, political and community mobilisation, and public-sector ART programs have meant that HIV is increasingly normalised in some important ways. Disclosure is still difficult but no longer rare. Politicians increasingly address the disease openly and even get tested in public. The notion of HIV infection as an automatic death sentence is weakening. This isn't to say that full normalisation has been achieved by any means--only that the social forms and interpretations of the disease have changed significantly in recent years.

While there is some evidence that HIV stigma is, overall, on the decline [10], stigma is poorly theorised and researched [11], making generalisations difficult. It is also important to keep in mind that changes in stigma have, and will continue to be, uneven and unpredictable. It may, in some settings, unexpectedly increase, even in the presence of accessible ART programs and community mobilisation. It can also take many forms, with one form of stigma fading as other, equally pernicious forms emerge [12]. Stigma can also affect different groups, like children or sex workers, in different ways [13] and require different strategies and interventions [14].

There have been important changes in the public health response to HIV as well. Shifts towards political and financial investments in ART programmes and health systems strengthening have meant that many governments are now committing to the mainstreaming, integration, and decentralisation of HIV care [15]. Not surprisingly, this process has also been uneven. The integration of HIV care into primary care services has enjoyed a range of critical successes in countries as varied as Brazil, the Dominican Republic and Zimbabwe, but it has also put enormous strain of many of these systems and exposed serious underlying weaknesses [16]. One response has been to shift tasks and de-professionalise HIV care by, for example, having nurses initiate ART on their own, allowing lay counsellors to do finger pricks as part of mass testing campaigns, and asking community health workers to serve as the front line of care provision. These changes reflect an increasingly popular model of HIV care and support that understands the disease as a long-term condition to be managed as much in the family and community as in the clinic [17,18].

Perhaps the most significant change, however, has been the scaling up of the ART programs in public sector health systems and the gradual but significant closing of the "treatment gap". In just one year, for example, between 2008 and 2009, ART coverage increased globally from 28% to 36% [19]. While still far short of what is needed, universal access to ART promises to be the key element in building public and political narratives that "things have changed", that HIV is at least on its way to no longer being a fatal acute disease but instead a manageable, long-term condition [20-22].

Thus, though HIV/AIDS was labelled a chronic disease as early as the late 1980s in the US, it has really only been in the last few years that it has been possible to use the language of chronicity to describe HIV in those parts of the rest of the world that have been hardest hit. But how might the global understandings of and responses to HIV change as a result of this growing interpretation of the epidemic as a chronic global condition? Many of the dramatic developments in the earlier history of the HIV epidemic were driven by a focus on HIV's acuity rather than its chronicity--its initially slow but consistently fatal progression, its remarkable ability to evade anti-retroviral treatments and vaccines, the significant stigma attached to it, and the scale of the epidemic. How will its emerging identity as a chronic disease with treatment options that dramatically extend life change how global actors understand and address HIV?

### What Will Chronicity Mean for the Global HIV Pandemic?

One thing is for certain: whether chronic or not, global economic forces will continue to structure in many ways the risks and vulnerabilities of people for HIV. This is not to say that the macroeconomic forces aren't changing. The global financial crisis has, for example, occasioned a certain degree of self-reflection and response to instabilities and inequalities in the global economic system. But the broad effects, both positive and negative, of economic globalisation and liberalisation, will continue to be felt in terms of both who gets infected and how those infected and affected by HIV are able to cope with the disease.

The economic vulnerabilisation of people, however, may also worsen as a result of the transformation of HIV into a chronic disease. On one hand, ART allows the most economically active portions of the population to return to work and this should ease the burden of coping with the disease. On the other hand, though, adherence challenges, episodes of serious illness, transaction and opportunity costs related to lifelong treatment, and the need for continued investment of public resources to fund treatment programmes will all put serious and sustained pressure on communities and states alike [23-25].

Global health governance and global health and development aid programmes will also face a number of new challenges. One will simply be maintaining the political support necessary for the scale of international funding required to manage HIV as a chronic condition. The recently stabilising incidence rates of HIV in Southern Africa along with the global financial crisis have raised intense concerns, for example, around the sustainability of global and national-level financing for ART programmes and other HIV prevention, care and treatment efforts [26-28]. On one hand, this outcry reflects a justifiable demand to maintain HIV as a global health priority and raises reasonable concerns around the fickleness of global health and development spending and the importance of maintaining targeted support in particularly vulnerable populations.

On the other hand, those who would critique the "AIDS industry" and the vested interests and habits of thinking that surround the disease do--conspiracy theories aside--have a point. Global funding for HIV has risen, for example, from around $300 million in 1996 to $13.7 billion as of 2009 [29], a massive increase but one that is still short of the real need. While this funding increase for HIV has taken place during a period of dramatic increases in global health and development funding overall, it remains the case that far more of this money is available for HIV than any other health condition [30]. The recent attention paid by the WHO to the neglect of non-communicable diseases (NCDs), for example, has cast current levels of HIV funding in stark contrast to NCDs which cause 80% of the deaths in developing countries but receive only 3% of global health development money [31].

The transformation of HIV into a chronic epidemic will thus entail both increased HIV-specific funding needs (especially as total treatment burdens increase and battles over intellectual property rights to second- and third-line treatment continue) as well as pressure to dislodge some of the institutional agendas, relationships, and resources that currently coalesce around the epidemic.

Debates around health funding involve not only questions of which diseases should get what money; they also ask whether disease-based funding is the best way to spend the money. There are already intense debates around the best forms of health development financing in an era of large-scale ART. The often polarised debates around verticalised programming versus horizontal programming and health systems strengthening will hopefully develop into more nuanced debates around, for example, "diagonal" approaches that both strike a balance between disease and systems priorities as well as use disease-specific interventions to leverage improvements in the broader health systems [32,33]. While some have cautioned that stripping HIV of its exceptional status will reverse the gains already secured [34], the integration of HIV services--along with the lessons of innovative HIV service delivery models--into other chronic and primary health care services has rightly been identified as a way to "jumpstart" improvements in the broader health system [35].

This integration also presents an important opportunity for AIDS activists to develop their strategies and join forces with the emerging political interest in the problems of NCDs and health systems. Working together, activists would be in a better position to push for long-term, sustained reform in health systems. Many are caught, however, within an increasingly competitive funding environment that still tends to reward those diseases that achieve the most visibility and urgency on the global scene, a dynamic that runs counter to equally important activist efforts to normalise HIV as a chronic disease.

There have been some interesting examples of NGOs and social movements working successfully across disease categories, addressing broader issues of health rights and social justice, and highlighting the social determinants of health. Social movements in South Africa like the Treatment Action Campaign (TAC) have been seeking out new territory and strategies in trying to determine what health activism will look like after widespread ART is available [36]. However, there haven't been many examples yet of AIDS activists joining together with others health activists groups and agendas. How HIV/health activism refigures and sustains itself in the face of widespread treatment is one of the most interesting questions about the current state of affairs.

For national health systems, thinking about HIV as a chronic condition entails a number of potentially dramatic changes. Some of the changes will be driven simply by scale. Closing the treatment gap described above will entail rapidly rising costs, not only for treatment but for diagnostic and monitoring tests, for counsellors, social workers and community health workers, for health information systems, and for health system infrastructure. These increases are, of course, in the context of competing health priorities (chronic and otherwise) and a likely persisting global economic malaise.

These changes will entail not only increases in the total amount of resources allocated to HIV but also to the organisation of the health system itself. Some form of integration and decentralisation of ART programmes, and HIV care more generally, will be necessary in many contexts. The scale of the necessary reorganisation and integration of health care services is potentially unprecedented, especially in the highest prevalence countries.

Scale, however, is not the only challenge for these large-scale, public sector programs. Complexity will also increase as the number of patients in long-term treatment increases. These complexities will be seen in long-term adherence challenges, resistance and treatment failures; co-morbidities with other conditions like diabetes, TB, cancer and dementia; and the intended and unintended interactions between treatment and prevention efforts [37,38]. While HIV may, therefore, fit the broad model of a chronic disease, it may also prove to be more complicated to prevent and treat than many other chronic diseases.

For those countries with smaller-scale epidemics and/or access to sufficient resources, many of these challenges can be addressed independently, at the national level. But for those countries without the resources to fully manage their epidemics, their choices will continue to be shaped by the broad range of global actors in HIV on whose support they will continue to rely as much as it will be by local contexts and resources [34]. Policymaking and decisions around health and development spending at the global level will therefore continue to have a powerful influence on how these countries are able to manage their epidemics.

### What Is Problematic About the Concept of Chronicity?

While the concept of chronicity has been productively used to describe and predict some of the recent transformations in the HIV epidemic, it is also not without its problems as a conceptual framework. Many of the conventional understandings of "chronic" disease--as diseases that are stable, manageable, and lifelong, as conditions that are invisible or at least without the usual acute signs, and as disorders linked to individual "lifestyles" and "behaviours"--do not adequately capture life with HIV for most people.

The critique and extension of the concept of the "chronic" is an area of active research in medical anthropology and elsewhere. The simple conceptual difficulties of maintaining the common acute-versus-chronic disease dichotomy (and the closely related infectious versus non-communicable disease distinction) have been well established in the early analyses of chronicity and acuity [39]. More recently though, this dichotomy has come under pressure for the ways it promotes an unrealistic, and indeed dangerous image of these diseases as stable, uniform, associated with "development" and old age, and manageable through simple technical interventions and individual agency (read compliance).

Consider, for example, the common narrative among activists, clinicians, public health researchers, and especially those infected with HIV, that anti-retroviral therapy has meant a singular resurrection from "near death" to "new life". These treatment narratives describe a dramatic transition from a state of personal, existential emergency to a state of good health and social reintegration, one where those with HIV aren't any different than anyone else [40,41]. Indeed, ART, for those who can get it and stay on it, can mean a radical transformation in the meaning and experience of HIV infection. And the expansion of public ART programmes represents a dramatic, qualitative shift in the epidemic. These treatment narratives have been critical in many countries in overcoming powerful denial and disbelief about the effectiveness of ARVs. AIDS activism has won a significant victory in this context in changing public opinion and state policies and securing dramatic gains in population health that ten years earlier seemed impossible.

However, it is also true that the conventional narratives of what acute and what chronic mean are inadequate for capturing these transformations, even under the best of circumstances. The narratives of chronic HIV infection and treatment described above centre on an image of either a resurrected body (the "Lazarus effect" of ART) or a vibrant, healthy body that never had to be resurrected (because of early treatment), a body that is strong and newly disciplined in maintaining treatment and lifestyle adherence, newly normalised as the sufferer, like billions of other people on the planet, of just another chronic condition with no specified endpoint.

What this narrative leaves out, however, are the sometimes dramatic fluctuations in health that characterise most chronic illnesses (and especially HIV). It ignores the fact that most chronic diseases are socially expected to be invisible and manageable and those who aren't seen to thrive sufficiently are stigmatised for this failure (the so-called "John Wayne" model of chronic disease [42]). It makes invisible the short and long-term physical toll and side effects of treatment and the considerable difficulty of maintaining adequate supplies and precise daily dosing of medication over the course of a lifetime that will for many also include unemployment, trauma, depression, and migration. Finally, treatment narratives that celebrate HIV's long-awaited arrival as a chronic condition mask the persistence of the local and global structural conditions that produced vulnerability and infection in the past and continued suffering and poor therapeutic adherence in the present.

In the end, those whose course of illness doesn't fit the model of stable, manageable, invisible chronic illness may come to be seen--by communities and by the health systems they rely on--either as "defaulters" or as unfortunate statistical outliers. And ART programmes grow, the number of people whose experiences of long-term treatment do not match with these high expectations will only increase.

As such, conventional discourses of chronicity can be a powerful constraint to our understanding of how HIV illness is produced, experienced and transformed. And this matters not only for individual experiences and interpretations of the disease. If used simplistically as a guiding conceptual framework for global health policy and programming around HIV, the idea of chronicity could prove similarly short-sighted and damaging. Just as HIV helped to catalyze a number of significant scientific, policy, and political developments beyond the epidemic itself, we should be using the opportunity of this latest phase of the epidemic to inspire shifts in our broader understandings of what "chronicity" means and how we should respond to it.

## Competing interests

The authors declare that they have no competing interests.

## Authors' contributions

CC conceived and drafted the article.

## Authors' Information

Christopher J. Colvin is Senior Research Officer in Social Sciences and HIV/AIDS, TB and STIs at the Centre for Infectious Disease Epidemiology and Research (CIDER) at the School of Public Health and Family Medicine at the University of Cape Town. His research interests include masculinity and HIV/AIDS, community mobilisation, global health activism and health citizenship around HIV/AIDS, the integration and decentralisation of primary health care, and the incorporation of qualitative and ethnographic methods into public health research and clinical trials.
